# Effects of Mechanical Loading on the Structure and Function of the Achilles Tendon: From Homeostatic Adaptation to Pathological Degeneration

**DOI:** 10.3390/jfmk11030273

**Published:** 2026-07-16

**Authors:** Linshu Guan, Weijian Zhang, Haoliang Wang, Yizhe Zhang, Jiachen Sun, Jun Lu

**Affiliations:** 1 School of Medicine, Southeast University, Nanjing 210009, China; gls20010517@163.com (L.G.); zelric@126.com (W.Z.); drwanghaoliang@163.com (H.W.); zhangyizhe2025@163.com (Y.Z.); 2 The Center of Joint and Sports Medicine, Orthopedics Department, Zhongda Hospital, School of Medicine, Southeast University, Nanjing 210009, China

**Keywords:** Achilles tendon, mechanical loading, tendinopathy, tendon healing, biomechanics, rehabilitation

## Abstract

The Achilles tendon, the largest and strongest tendon in the human body, exhibits dynamic adaptive changes in its structure and function through mechanobiological regulation. This review synthesizes the dual regulatory effects of mechanical loading on Achilles tendon homeostasis and pathology: Moderate mechanical stimulation activates integrin-mediated signaling pathways (including PI3K/Akt and MAPK/ERK cascades), promoting tenocyte proliferation/differentiation, collagen biosynthesis, and orderly remodeling of extracellular matrix (ECM), thereby enhancing tendon stiffness, elastic modulus, and ultimate tensile strength. Conversely, chronic overload or disuse conditions induce collagen disorganization, aberrant matrix metalloproteinase (MMP) expression, and inflammatory cascades, creating a predisposition to tendinopathy and degenerative disorders. Emerging evidence highlights the critical role of mechanotransduction in injury repair, with early-stage progressive loading regimens demonstrating enhanced healing outcomes through optimized ECM metabolism and biomechanical signal propagation. Clinically, individualized load management strategies, including blood flow restriction training and biomaterial-assisted mechanomodulation, show promise in injury prevention and rehabilitation. Future research integrating multi-omics approaches with intelligent load-monitoring technologies may clarify mechanobiological coupling mechanisms and facilitate precision interventions for Achilles tendon disorders.

## 1. Introduction

The Achilles tendon is one of the largest and strongest tendons in the human body [1], and its function is to transmit the force generated by the gastrocnemius muscle to the foot [2], enabling activities such as walking and running. The histological composition of the Achilles tendon is primarily type I collagen, and these collagen fibers are arranged in parallel bundles, with tendon cells distributed parallel to these fibers. The collagen fibers further aggregate into primary, secondary, and tertiary fiber bundles, ultimately forming tendon units that exhibit a highly organized structure, providing the Achilles tendon with excellent mechanical strength and elasticity [3]. However, anatomically, the vascular distribution of the Achilles tendon shows a characteristic of “more at the ends, less in the middle,” with the midsection being more prone to injury, making repair after damage more difficult.

Mechanical loading is a key factor affecting the structure and function of the Achilles tendon. Under physiological conditions, the mechanical signals generated by mechanical loading stimulate tendon cells to self-regulate and remodel the ECM, thereby maintaining the normal functional state of the tendon tissue [4]. However, excessive or insufficient mechanical loading can lead to disruption of Achilles tendon structure and dysfunction, further resulting in tendon tissue damage [5]. Studies have shown that long-term load imbalance in the Achilles tendon may lead to tendinopathy, a condition characterized by reduced mechanical performance and functional impairment, which impacts the quality of life of patients [6]. Moderate mechanical stimulation can play a positive role in the repair after Achilles tendon injury or the maintenance of its physiological function. Therefore, studying how mechanical loading affects the physiological and pathological processes of the Achilles tendon, and understanding the mechanisms that maintain tendon structure and function under mechanical load is of significant importance for formulating prevention and treatment strategies for Achilles tendon diseases.

This review aims to summarize how mechanical loading influences the structure and function of the Achilles tendon. It specifically explores the underlying mechanobiological mechanisms that drive tendon tissue’s adaptive responses and homeostatic maintenance, providing a comprehensive synthesis of current evidence. By reviewing existing literature, this review elucidates the regulatory role of mechanical loading on tendon cell metabolism, collagen synthesis, and changes in histological structure, along with the underlying molecular mechanisms. Additionally, this review will summarize the relationship between different loading conditions and pathological changes in the Achilles tendon, as well as potential therapeutic strategies, including the optimized application of load management in Achilles tendon injury repair. Ultimately, this review aims to bridge the gap between basic mechanobiology and clinical practice, providing orthopedic surgeons and rehabilitation specialists with evidence-based insights for optimizing load management in the prevention and treatment of Achilles tendon disorders.

Going beyond macroscopic biomechanics, this review highlights the crucial interplay between aberrant mechanotransduction and pathological microenvironments. By integrating recent single-cell multi-omic findings, we elucidate how dysregulated loading and damage-associated molecular patterns (DAMPs) synergize to drive fibrotic repair. Furthermore, we translate these molecular insights into clinical practice, exploring how metabolic comorbidities alter tendon mechanosensitivity and necessitate individualized load management strategies.

## 2. The Structure and Function of the Achilles Tendon

Anatomically, the Achilles tendon is the largest and strongest tendon in the human body. It is primarily composed of the gastrocnemius and soleus muscles, which merge approximately 15 cm above the heel and attach to the calcaneal tuberosity [7]. Importantly, the plantaris tendon also courses parallel to the medial aspect of the Achilles tendon. Its anatomical proximity and potential to exert compressive or shearing forces are now recognized as critical factors in the pathobiology of mid-portion tendinopathy [8]. Furthermore, the Achilles tendon exhibits a characteristic twisted structure. While often described as an internal rotation of up to 90° that optimizes mechanical efficiency during joint movement [9], recent studies emphasize that the degree of this twist displays significant inter-individual variation and depends on measurement methodology [10]. Despite its relatively slender morphology, the tendon’s high extensibility allows it to maintain optimal biomechanical performance during foot movements [11].

The histological structure of the Achilles tendon is similar to that of other tendons, with densely arranged fibers as the main component. The primary collagen type in the Achilles tendon is type I collagen (accounting for approximately 65–80% of the dry weight), with a small amount of type III collagen [3]. Type I collagen forms collagen fibrils, which then aggregate to form collagen fibers. In healthy Achilles tendons, collagen fibers are arranged longitudinally, forming parallel fiber bundles [12]. Regional variations in collagen fiber diameter and the presence of an elastin-rich endotenon allow for independent interfascicular sliding, which is essential for the tendon’s functional adaptation to multi-axial mechanical loads. The ECM, in addition to collagen, includes various collagen isoforms, glycoproteins, proteoglycans, and water. Compared to other tissues, tendons have relatively few cells, but they still contain various cell types, such as endothelial cells, pericytes, nerve cells, erythrocytes, and immune cells [13]. Tenocytes, a highly specialized form of fibroblast, constitute the vast majority of this cellular population—typically accounting for 90% to 95% of the cells in healthy tendon tissue [14]. Their cell bodies extend multiple thin, wing-like protrusions that embed between the fiber bundles. Tendon cells can synthesize high levels of collagen and also maintain the structural integrity of Achilles tendon tissue by regulating ECM components. The Achilles tendon lacks a tendon sheath but is encased by a highly vascularized paratenon [15]. The paratenon serves as a conduit for tendon blood vessels and facilitates the movement of the tendon between the subcutaneous tissue and fascia. Studies have revealed that mechanical loading dynamically balances tendon cell function and collagen metabolism through Ca^2+^, BMP, and other signaling pathways in a spatiotemporal-specific manner [16]. This bidirectional regulatory mechanism may enhance mechanical properties by promoting collagen cross-linking, but it also carries the risk of abnormal remodeling (such as ectopic calcification), jointly determining the adaptive outcomes of the Achilles tendon [17,18].

The blood supply of the Achilles tendon is mainly derived from the posterior tibial artery and the peroneal artery, with the peroneal artery supplying the mid-portion and the posterior tibial artery supplying the remaining proximal and distal regions; however, the overall vascular density is limited. Heterogeneous vascular distribution is another characteristic of the Achilles tendon, with vessels predominantly concentrated at the myotendinous junction and the osteotendinous junction, whereas the mid-portion exhibits relatively sparse vascularity [19], which may result in local hypoxia and metabolic disturbances and thereby increase the risk of degenerative changes and rupture. The inherently limited regenerative capacity of the Achilles tendon is attributed, in part, to its relatively hypovascular environment, alongside other biological and mechanical constraints that govern the healing process.

**Figure 1:**
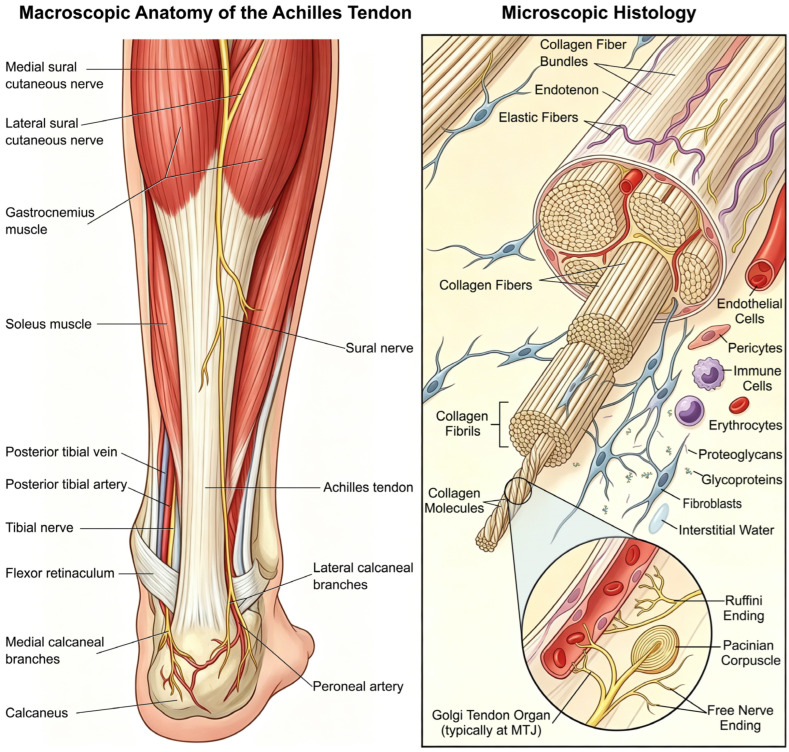
Multiscale anatomical and histological characteristics of the Achilles tendon. (**Left**) Macroscopic Anatomy: The Achilles tendon is formed by the confluence of the gastrocnemius and soleus muscles, attaching distally to the calcaneal tuberosity. The vascular supply exhibits a heterogeneous distribution, predominantly derived from the posterior tibial and peroneal arteries, with a relatively avascular zone in the mid-portion (a common site for rupture). Innervation is primarily supplied by the sural nerve. (**Right**) Microscopic Histology: The tendon exhibits a hierarchical organization where Type I collagen fibers (predominant structural component) aggregate to form fascicles surrounded by the endotenon. Tenocytes are interspersed between collagen bundles, extending processes to sense mechanical signals. The matrix also contains elastin, proteoglycans, and minor collagen types (Type III). The neurovascular network includes sensory receptors (e.g., Pacinian corpuscles, Ruffini endings) that mediate proprioception and nociception.

The Achilles tendon is primarily innervated by nerve fibers originating from the sural nerve, which courses along the lateral aspect of the tendon. The sural nerve is formed by the convergence of branches from the tibial nerve and the common peroneal nerve and passes along the lateral side of the Achilles tendon approximately 8–10 cm proximal to the superior border of the calcaneus [20]. In addition, sensory receptors associated with the Achilles tendon exhibit distinct spatial distributions. While Ruffini endings (for pressure perception), Pacinian corpuscles (involved in motor regulation), and free nerve endings (responsible for pain sensation) are distributed within the tendon proper and its surrounding tissues [21], Golgi tendon organs, which detect changes in tension, are classically restricted to the myotendinous junction rather than being distributed throughout the main body of the tendon [22]. Previous studies have demonstrated that these neural components are closely associated with vascular structures within the tendon tissue and the surrounding connective tissue under tendinopathic conditions [23]. Figure 1 illustrates the macroscopic anatomy, hierarchical collagen organization, and neurovascular supply of the Achilles tendon. Understanding these multiscale anatomical features is of profound clinical relevance. For instance, the relative avascularity of the mid-portion depicted structurally predisposes this region to delayed healing and an increased risk of degenerative tendinopathy or spontaneous rupture. Furthermore, the precise distribution of sensory nerve endings provides a crucial anatomical basis for understanding the neurovascular ingrowth and nociceptive pain characteristic of chronic tendinopathy.

The Achilles tendon directly transmits the force generated by muscle contraction to the foot, thereby driving plantarflexion and producing the propulsive force required for walking, running, and jumping. During gait, both the gastrocnemius and soleus muscles serve as the primary plantarflexors at the ankle joint. These muscles are highly active during the final 80% of the stance phase of walking and play a critical role in propulsion and balance. Recent studies have examined the contribution of individual muscles to ankle plantarflexion force and have shown that the soleus contributes more during slow walking, whereas the gastrocnemius plays a more prominent role during fast running [24]. Although the medial and lateral heads of the gastrocnemius have similar plantarflexion functions, the medial head contributes more than 70% of the total force. The Achilles tendon exhibits a tensile strength of several thousand Newtons and an extensibility of approximately 6% to 8% [25], and these superior mechanical properties enable optimization of energy efficiency during the storage and release of elastic potential energy [26]. In addition, the Achilles tendon regulates mechanical loading through adaptive responses, ensuring the maintenance of structural stability and functional integrity under various movement conditions [27].

## 3. Types of Mechanical Loading

Mechanical loading can be classified according to loading pattern, duration, and intensity, with different types of loading exerting distinct biological and mechanical effects on tendon tissue stiffness.

### 3.1. Dynamic Loading and Static Loading

Mechanical loading applied to the Achilles tendon can be classified into dynamic and static loading, each of which exerts distinct effects on tendon structure and function. Dynamic loading refers to cyclic mechanical stress applied to the tendon, typically characterized by alternating phases of tension and relaxation. Common movement patterns involving dynamic loading include walking and running, through which dynamic loading stimulates tenocytes and the ECM to undergo adaptive changes that enhance tendon elasticity and strength. Studies have shown that dynamic loading upregulates type I collagen–related genes by approximately tenfold via mechanotransduction and induces anabolic metabolic changes in tenocytes [5], thereby contributing to the maintenance of normal tendon physiological function and prompting its use in investigations of tendon repair mechanisms. In contrast, static loading refers to constant mechanical stress applied to the tendon, typically manifested as sustained tension under a fixed stretched state. During daily activities and rehabilitation training, individuals are often required to maintain specific postures, under which the mechanical stimulus experienced by the tendon constitutes static loading. Static loading does not induce cyclic variations in tension but can lead to collagen fiber remodeling and redistribution of tensile forces within tendon tissue. Evidence suggests that sustained static loading can increase tendon stiffness and optimize collagen fiber alignment to improve mechanical properties [28], although its stimulatory effect on collagen synthesis is relatively limited [29].

### 3.2. Acute Loading and Chronic Loading

Acute loading refers to high-intensity mechanical stress applied to the tendon over a short period of time and is commonly observed during strenuous physical activity. Acute loading is characterized by high intensity and short duration and can exert significant effects on tendon structure and function, such as rapidly altering tendon stiffness and energy storage capacity. Consequently, acute loading has been widely used in research to assess tendon tolerance [30]. In contrast, chronic loading represents the accumulation of repetitive low- to moderate-intensity mechanical stress over extended periods, typically lasting from weeks to months. Chronic loading mainly arises from daily activities. The effects of chronic loading on tendon tissue are primarily reflected in adaptive responses, including changes in mechanical properties, morphological characteristics, and material properties [6]. Kubo et al. reported that after three months of resistance training, Achilles tendon stiffness increased by an average of 50.9%, based on measurements of tendon properties and metabolic changes during training and detraining periods [31]. During the repair of Achilles tendon injuries, appropriate chronic loading is beneficial for restoring biomechanical properties, whereas prolonged excessive chronic loading may lead to degenerative changes in the tendon [32].

### 3.3. High-Intensity, Excessive, and Moderate Loading

To maintain scientific consistency and clinical applicability, it is essential to operationalize the mechanical loading parameters discussed throughout this review. Recent systematic reviews quantifying Achilles tendon forces emphasize that loading magnitude can be effectively categorized using multiples of body weight (BW) [33].

High-intensity loading is typically characterized by mechanical strains that approach or exceed the physiological limits observed during maximal dynamic activities. Clinically, this is generally defined as loads exceeding 4.0 BW, corresponding to activities such as running, plyometric hopping, or heavy single-leg heel raises (e.g., with additional load >125% BW). While in vivo studies have reported Achilles tendon strains ranging from 6.0% to 9.0% during running and jumping [33], these values exhibit significant variability depending on the specific task, movement velocity, and individual biomechanical profiles [10,34]. This type of loading typically occurs during strenuous exercise or high-intensity training and is characterized by high magnitude, short duration, and elevated peak stress, enabling rapid increases in tendon stiffness and strength.

In contrast, moderate- and low-intensity loading corresponds to loads typically below 4.0 BW, encompassing activities like walking (2.7 to 3.95 BW) or foundational rehabilitation exercises. This level of loading generally remains within the tendon’s physiological tolerance and is well below the threshold for rupture. Therefore, low-intensity loading is more suitable for early-stage rehabilitation following Achilles tendon injury to avoid secondary damage.

Furthermore, excessive loading is explicitly defined pathologically as a state where matrix damage accumulates because recovery time is insufficient and/or the tendon has an impaired adaptive capacity. Evidence suggests that excessively high-intensity single loading bouts can increase the expression of tendinopathic markers, such as inflammatory cytokines and degenerative enzymes, including MMPs and prostaglandin E2, thereby inducing collagen fiber damage within the tendon [35]. By monitoring pre-competition training loads in athletes, Esmaeili et al. observed that periods of high-intensity loading were associated with structural alterations in the Achilles tendon, such as increased thickness and changes in matrix organization. These observational findings suggest that while intense loading correlates with structural remodeling, timely reduction of load intensity—preventing it from becoming excessive—may allow for the stabilization of these adaptive changes [36].

## 4. Effects of Mechanical Loading on the Structure and Mechanical Properties of the Achilles Tendon

Mechanical loading is a key factor regulating the structure and mechanical properties of the Achilles tendon. Appropriate mechanical stimulation promotes collagen synthesis and ECM remodeling, increasing the cross-sectional area (CSA), stiffness, and elastic modulus of the Achilles tendon and thereby enhancing its load-bearing capacity, whereas excessive or insufficient loading disrupts collagen organization, suppresses adaptive remodeling, and leads to deterioration of tendon mechanical properties [37].

### 4.1. Effects on Structure

Mechanical loading exerts profound effects on the morphological structure and macroscopic characteristics of the Achilles tendon. Under moderate loading conditions—defined as submaximal locomotive tasks or cyclic loading at 40–60% of maximum voluntary isometric contraction—the tendon adapts through collagen synthesis and ECM remodeling [38]. These adaptations include increases in CSA and task-specific changes in subtend on length, the latter of which are primarily attributed to non-uniform interfascicular sliding, ultimately enhancing tensile strength and mechanical efficiency. After resistance training in participants of different age groups, Adrien et al. assessed Achilles tendon size using magnetic resonance imaging and evaluated mechanical properties via ultrasonography and tendon mechanical testing, finding significant increases in tendon stiffness, Young’s modulus, and CSA regardless of age [39]. From a gross anatomical perspective, the three subtendons of the Achilles tendon exhibit a twisted configuration, resulting in differential morphological changes among subtendons when mechanical loading is applied. Knaus et al. developed a three-dimensional finite element model of the human Achilles tendon and demonstrated that fiber-aligned strain within subtendons decreases with increasing degrees of twist [40]. Excessive mechanical loading, however, can adversely affect the macroscopic tendon structure, leading to disorganized fiber alignment, increased vascularization, and tissue edema, which are commonly indicative of the onset or progression of tendinopathy. In contrast, insufficient mechanical loading suppresses anabolic signaling in tenocytes, resulting in reduced total collagen content in the ECM, disorganized fiber arrangement, and inadequate functional cross-linking. This maladaptive remodeling ultimately manifests macroscopically as reductions in Achilles tendon CSA, stiffness, and elastic modulus, thereby markedly compromising its mechanical performance [41].

Collagen fibers are the principal determinants of the mechanical properties of the Achilles tendon, and their organization and biological activity are directly regulated by mechanical loading. First, the application of mechanical loading, particularly high-intensity or repetitive loading, can induce alterations in collagen fiber alignment as well as changes in the quantity and quality of intrafibrillar cross-linking. For example, Takahashi et al. reported that under mechanical loading, the Achilles tendon enthesis transfers load from soft tissue to hard tissue via fibrocartilage, whereas load deprivation induces histological changes in collagen organization, manifested by a significant reduction in the parallel alignment of collagen fiber bundles [42]. In addition, Connizzo et al. demonstrated in ex vivo animal experiments that under diabetic conditions, structural remodeling of Achilles tendon collagen fibers begins at relatively low loading levels and varies significantly across regions, ultimately resulting in reductions in stiffness and elastic modulus [43]. Second, consistent with most tendons, type I collagen is the most critical structural protein in the Achilles tendon and determines its tensile strength and adaptive capacity. Evidence indicates that moderate dynamic mechanical loading can stimulate fibroblast activity in tendon tissue via mechanotransduction pathways, enhancing the synthesis and secretion of type I collagen and thereby optimizing the tensile strength and stiffness of the Achilles tendon [44].

### 4.2. Effects on Stiffness and Elasticity

Stiffness and elasticity are core parameters for evaluating the biomechanical properties of tendons. Stiffness reflects the tendon’s resistance to elastic deformation during stretching, whereas elasticity represents its ability to return to its original length after elongation. Studies have shown that sustained high-intensity loading can significantly increase tendon stiffness, thereby improving the efficiency of force transmission from muscle to skeleton [45]. As with most tendons, stiffness and elasticity also determine the Achilles tendon’s responsiveness to mechanical loading. Using real-time ultrasonography, Maganaris et al. demonstrated that mechanical loading-based training can significantly increase Achilles tendon stiffness. This adaptation may optimize athletic performance by enhancing the tendon’s capacity to store and release elastic energy [40]. Moreover, different training modalities exert distinct effects on Achilles tendon stiffness and elasticity, and a systematic review by Lazarczuk et al. revealed that resistance training produces more pronounced improvements than other training types [46]. In addition to training modality, factors such as loading cycle duration and load intensity also contribute to varying degrees of improvement in Achilles tendon stiffness and elasticity. A systematic review by Wiesinger et al. indicated that increasing load intensity and prolonging physical training duration can significantly enhance Achilles tendon stiffness and elasticity [47]. As shown in Table 1, previous studies have indicated that different exercise modalities and frequencies result in distinct changes in the mechanical properties and structure of the Achilles tendon. From a clinical perspective, these findings provide an evidence-based framework for tailoring rehabilitation prescriptions to individual patient capacities. For example, while high-strain isometric (ISO) and stretch-shortening cycle (SSC) protocols are highly effective for optimizing tendon stiffness in healthy athletes or during late-stage rehabilitation, low-load blood flow restriction (LL-BFR) training emerges as a crucial clinical alternative. Because LL-BFR induces comparable hypertrophic and mechanical adaptations at significantly lower strains, it represents an exceptionally valuable strategy for patients who cannot safely tolerate high mechanical loads early in their recovery process.

### 4.3. Effects on Modulus

Young’s modulus is an intrinsic material property that quantifies the inherent resistance of the tendon matrix to elastic deformation, independent of its geometric dimensions. Unlike structural stiffness—which describes the force–displacement relationship of the whole tendon and is influenced by its CSA and length—the modulus is derived from the macroscopic stress–strain relationship within the linear elastic region [53]. While the modulus is determined through macroscopic mechanical testing, it reflects the underlying quality and hierarchical organization of the collagenous ECM. Consequently, mechanical loading can modulate Achilles tendon function through two distinct pathways: by altering macroscopic structural stiffness via hypertrophic changes and by inducing material-level alterations in the tendon modulus. In general, researchers design specific experimental protocols to apply different loading regimens and observe the resulting changes in the Achilles tendon. Létocart et al. used ultrasonography to evaluate the biomechanical properties of the patellar tendon and Achilles tendon in 27 healthy elderly men before and after training with different load intensities. The results showed a significant increase in the Young’s modulus of the Achilles tendon following training. Notably, high-intensity loading produced a more pronounced increase in Young’s modulus, with an average improvement of 26.1%, demonstrating that appropriate mechanical stimulation can promote tendon adaptation and enhance elastic modulus [54]. However, excessive or improper loading can lead to a reduction in the Young’s modulus of the tendon. Particularly after the occurrence of microstructural damage, a decrease in modulus increases tendon brittleness and elevates the risk of Achilles tendon injury. For example, experimental evidence indicates that short-term overtraining may reduce Young’s modulus, accompanied by disruption of structural integrity [55]. Additionally, Camy et al. characterized the micromechanical properties of the murine Achilles tendon enthesis using microindentation testing and Raman spectroscopy, finding that simulated unloading had no significant effect on elastic modulus, whereas reloading after unloading led to a marked decrease in modulus [56], indicating that the absence of mechanical loading may weaken the tendon’s resistance to deformation and indirectly impair its function.

## 5. Effects of Mechanical Loading on Cells and Molecular Biology

Mechanical loading is a key regulator of Achilles tendon homeostasis and adaptation. Physiological loading promotes tenocyte proliferation, differentiation, and ECM remodeling through mechanotransduction, whereas insufficient or excessive loading disrupts these processes and contributes to tendinopathy. Mechanical signals are sensed by integrins and transmitted via pathways such as MAPK/ERK and PI3K/Akt, leading to load- and time-dependent regulation of tendon-related genes, including *SCX*, *TNMD*, *Egr1*, and collagens [57].

### 5.1. Effects of Loading on Tenocytes: Proliferation and Differentiation and ECM Synthesis and Degradation

Mechanical loading exerts significant effects on tenocytes. First, moderate mechanical stimulation not only promotes tenocyte proliferation but also induces their differentiation into specialized tendon cells that play a critical role in maintaining tendon structure and function. Multiple studies have indicated that mechanical stimulation is crucial for enhancing tenogenic differentiation of pluripotent stem cells, bone marrow stromal cells, bone marrow-derived stem cells, and tendon stem/progenitor cells (TSPCs) [58,59,60,61]. Second, mechanical loading also affects the composition and metabolism of the ECM in Achilles tendon tissue. Studies have shown that in tendinopathic states induced by excessive loading, the expression of type I collagen, type III collagen, MMPs, and other ECM proteins is higher in the Achilles tendon ECM than in healthy tendons [62,63]. Moreover, Grinstein et al. demonstrated that under mechanical stimulation, Achilles tenocytes synthesize and secrete greater amounts of ECM components, thereby promoting tendon repair and remodeling [64]. The process by which tenocytes experience mechanical stimulation involves complex cellular responses and adaptive mechanisms informed by external mechanical cues, prompting extensive in vivo and in vitro studies to further elucidate the specific effects of mechanical loading on tenocytes. In in vitro experiments, researchers commonly use cell stretching protocols to apply cyclic mechanical strain to human- or murine-derived tenocytes, thereby simulating the mechanical environment experienced during human movement [65]. Conversely, in vivo experiments alter the mechanical environment in animal models by increasing or decreasing load levels at specific sites, allowing a more comprehensive investigation of the effects of mechanical loading on tenocytes. In 2020, Fleischhacker et al. conducted a comparative study examining the effects of in vivo and in vitro mechanical loading on tenocytes and found that in vitro models are suitable for investigating early cellular responses to mechanical stimulation, such as nuclear morphology and selected signaling pathways, but require optimization through approaches such as three-dimensional culture and dynamic ECM scaffolds. Mechanisms involving ECM remodeling, systemic inflammation, or long-term adaptive changes, however, still rely on in vivo experimentation [66].

### 5.2. Signal Sensors and Transduction Molecules

Tenocytes play a pivotal role in maintaining tendon structure and function, and mechanical loading regulates tenocyte behavior through multiple signal transduction pathways. At the microscopic level, mechanotransduction promotes tenocyte adaptation to diverse mechanical environments, while at the macroscopic level it ensures that tendons can withstand repetitive loading and adapt effectively [67], forming the basis for tendon health. Recent studies have identified multiple signal sensors, transduction molecules, and mechanotransduction pathways, providing an important mechanistic basis for understanding tenocyte biology and guiding strategies to promote repair after tendon or ligament injury [68,69]. Mechanotransduction is initiated at the cell membrane, where mechanosensitive receptors such as integrins form complexes with focal adhesion proteins, including talin, vinculin, and paxillin, thereby establishing a physical linkage between the ECM and the actin cytoskeleton [70]. This mechanotransduction mechanism transmits signals across the membrane to directly activate key intracellular signaling cascades, such as the MAPK/ERK, Rho/ROCK, and PI3K/Akt pathways, thereby regulating cellular function through modulation of gene expression, cytoskeletal remodeling, and post-translational protein modifications [71].

Integrins are a class of essential transmembrane receptors that not only mediate adhesion between the intracellular actin cytoskeleton and the ECM but also serve as primary sensors of mechanical stimuli, playing a key role in converting mechanical loads into intracellular biochemical signals. The integrin α1 and α2 subunits are widely expressed [72,73], whereas integrin α11 is specifically localized around dense type I collagen and binds tightly to it [74]. Studies have shown that the integrin α11 promoter contains consensus sequences for binding the tendon-specific transcription factor SCX [74], and that mechanical loading applied to tendon stem cells increases the expression of multiple integrin subtypes, including α1/2/11, while activating downstream kinases such as ERK and p38 [75], indicating the involvement of integrins in tendon mechanotransduction. Evidence indicates that under mechanical stimulation, integrins recruit focal adhesion kinase (FAK) and SRC kinase, thereby initiating downstream signaling cascades that lead to phosphorylation of transcriptional regulators such as YAP/TAZ and ultimately modulate the expression of genes involved in cell proliferation, differentiation, and ECM remodeling [76].

### 5.3. Signaling Pathways

As described above, mechanical stimuli activate key intracellular signaling pathways through transmembrane signal transmission, including MAPK/ERK, PI3K/Akt, and Akt/mTOR pathways, thereby driving gene expression in tenocytes and ECM remodeling. Insulin-like growth factor 1 (IGF-1) is one of the earliest molecules identified to play a role in load-induced tendon hypertrophy. Current studies indicate that exogenous IGF-1 application in vitro activates the PI3K/Akt and ERK signaling pathways, thereby promoting protein synthesis and proliferation in tenocytes [77]. The MAPK/ERK pathway can also be directly activated by mechanical loading and plays a role in increasing collagen content; ERK signaling can be activated within 10 min of mechanical loading [78], and intermittent loading optimized for ERK1/2 phosphorylation increases collagen content more effectively than continuous loading. However, the mechanisms by which ERK increases collagen content are not fully understood; some studies suggest that ERK may enhance collagen production via the transcription factor EGR1, which regulates *COL1A1* [79]. Another pathway directly activated by mechanical signals in tenocytes is the PI3K/Akt signaling pathway. Crucially, the biological outcome of PI3K/Akt activation is highly magnitude-dependent and dictated by specific mechanical thresholds and tissue states [53]. Under physiological loading conditions, this pathway integrates mechanical stimuli with biochemical signals and plays an important role in tendon homeostasis and repair. First, moderate mechanical stimulation activates the PI3K/Akt pathway to regulate tenocyte proliferation and survival; Disser et al. demonstrated that PI3K/Akt signaling enhances tenocyte proliferation and inhibits apoptosis by modulating downstream factors such as mTOR and GSK-3β, thereby maintaining tendon structure and function [77]. Second, this pathway plays a critical role in the differentiation of tendon progenitor cells into mature tenocytes. Studies have shown that curcumin can promote the differentiation of TSPCs into tenocytes by activating the PI3K/Akt pathway, thereby enhancing tendon repair capacity [80]. Conversely, when mechanical loading exceeds the physiological safety threshold—such as during acute overloading or sustained excessive friction—the mechanobiological response diverges. In these pathological tissue states, the PI3K/Akt/mTOR axis becomes hyperactivated, shifting the cellular balance from anabolism to catabolism [53]. For instance, Yao et al. reported that excessive mechanical friction activates the PI3K/Akt/mTOR pathway, leading to overexpression of MMP-13 and reduced type I collagen synthesis, ultimately resulting in matrix degradation and fibrotic scar formation [81]. Therefore, the PI3K/Akt pathway acts as a mechanosensitive rheostat, directing the tendon toward either homeostatic adaptation or pathological degeneration depending on the intensity and nature of the applied load [82].

### 5.4. Regulation of Gene Expression by Mechanical Loading

Mechanical loading exerts a bidirectional regulatory effect on tenocyte gene expression, promoting tendon homeostasis and repair under physiological conditions while contributing to tendinopathy when loading is excessive or insufficient. Mechanical stimulation facilitates tenogenic differentiation and gene expression, particularly of tendon-specific markers such as scleraxis (*SCX*) and tenomodulin (*TNMD*). Cyclic mechanical stress has been shown to upregulate these genes, thereby enhancing tenocyte differentiation and supporting the formation of a load-bearing collagen type I-dominant framework, while also regulating the proteoglycan and elastin content essential to the tendon structure [83]. Mechanical loading also modulates genes involved in ECM metabolism. Type I and type III collagens constitute the primary structural components of tendon tissue, and moderate mechanical stimulation preferentially increases *COL1* expression. In contrast, excessive mechanical stress may induce *COL3* upregulation, thereby compromising tendon mechanical properties [84]. Overloading has additionally been associated with increased expression of MMPs, resulting in ECM degradation and structural weakening [81]. Conversely, appropriate mechanical loading enhances tendon repair by accelerating matrix protein synthesis and improving tenocyte survival [85]. Importantly, mechanically induced gene responses exhibit pronounced temporal dependency and molecular specificity. For example, Huang et al. isolated primary tenocytes from rat Achilles tendons and applied cyclic biaxial stretching using a Flexcell system, showing that short-term mechanical loading significantly upregulated early *EGR1* expression, while *SCX*, *MKX*, and collagen expression remained unchanged [86]. Thus, EGR1 expression is more sensitive to short-term mechanical stretching than other genes. While these in vitro findings highlight EGR1 as a mechanosensitive candidate, it is crucial to recognize that 2D biaxial stretching of isolated rat tenocytes lacks the complex 3D ECM interactions and systemic biochemical cues present in the human in vivo environment. Further clinical validation is required to confirm this pathway in human tendinopathy.

### 5.5. Single-Cell Insights and Fibroblast–Immune Crosstalk

Recent advances in single-cell RNA sequencing (scRNA-seq) have significantly expanded our understanding of the cellular microenvironment in human Achilles tendinopathy, shifting the focus toward fibroblast heterogeneity and immune interactions [87]. While physiological loading promotes ECM homeostasis, dysregulated mechanical loading synergizes with tissue microdamage to release DAMPs and alarmins, such as high-mobility group box 1 (HMGB1) and the S100 protein family [88]. This biomechanical-biochemical crosstalk triggers sustained inflammatory cascades via NF-κB and p38 MAPK pathways. Furthermore, aberrant mechanotransduction promotes the transition of resting tenocytes into activated fibroblast subpopulations expressing markers such as PDPN, THY1, and FAPαp [89,90]. These activated fibroblasts orchestrate intense fibroblast-immune cell crosstalk, actively recruiting CD45+ immune cells (including macrophages and T cells) and releasing pro-inflammatory cytokines and chemokines. Consequently, this sustained inflammatory loop disrupts normal matrix remodeling, driving the Achilles tendon toward pathological fibrotic repair rather than functional regeneration.

## 6. Mechanical Loading and Achilles Tendon Injury

Mechanical loading plays a central role in the development of Achilles tendon injury, and both acute and chronic injuries are closely associated with the loads applied to the tendon. Both excessive and insufficient loading can promote pathological changes in the Achilles tendon, including reduced mechanical properties, disorganized collagen alignment, and diminished adaptive capacity [91,92]. Therefore, maintaining appropriate loading intensity and frequency is essential for preserving Achilles tendon health and preventing injury.

### 6.1. Acute Achilles Tendon Injury

The Achilles tendon plays a critical role in force transmission during movement and is therefore highly susceptible to acute injury. Acute Achilles tendon injuries are typically caused by excessive mechanical loading or external impact over a short period, most commonly presenting as tendon rupture or tearing. In recent years, the incidence of Achilles tendon rupture has increased, likely due to population aging, rising obesity rates, and greater participation in sports [93]. Mechanical loading plays a dual role in Achilles tendon physiology, serving as a necessary stimulus for tissue homeostasis and adaptation, while excessive or inappropriate loading can precipitate tendon rupture. Acute Achilles tendon rupture most commonly occurs during high-impact activities involving rapid changes in loading, such as sprinting, jumping, or sudden directional changes. Studies have shown that during intense exercise, peak Achilles tendon strain can reach approximately 12%, approaching its failure threshold and thereby increasing rupture risk [94]. Freedman et al. demonstrated that fatigue loading significantly compromises tendon structural integrity, with accumulated microtears predisposing the tendon to acute rupture [95]. In addition, Bojsen-Møller et al. reported that non-uniform mechanical load distribution across different regions of the Achilles tendon leads to local stress concentrations, further elevating rupture risk [32]. Excessive mechanical loading can also induce physiological changes that impair the mechanical properties of the Achilles tendon. Previous studies have shown that excessive loading upregulates MMP3 and MMP13 expression, resulting in collagen degradation and reduced tendon strength [96]. Hammerman et al. demonstrated that high mechanical loading activates pro-inflammatory cytokines, accelerates tendon degeneration, and impairs repair capacity, thereby increasing the risk of rupture following load-induced microtears [97].

### 6.2. Chronic Achilles Tendon Injury

Chronic Achilles tendon injury, commonly referred to as Achilles tendinopathy, is a disabling overuse injury that is particularly prevalent among athletes. Achilles tendinopathy most commonly affects the mid-portion of the tendon and is clinically characterized by pain, swelling, and restricted mobility [98]. Epidemiological data estimate the lifetime risk of Achilles tendinopathy to be 52.0% in elite athletes and 23.9% in recreational athletes [99]. Studies have demonstrated that excessive mechanical loading is a major contributing factor in the development of Achilles tendon pathology. Under conditions of repetitive use and mechanical overload, the Achilles tendon exhibits severe collagen disorganization, abnormal deposition of type III collagen, and failure of reparative signaling such as inducible nitric oxide synthase (iNOS), ultimately resulting in characteristic tendon degeneration [100]. First, existing evidence indicates that prolonged excessive mechanical stress leads to the accumulation of microdamage, ECM degradation, and inflammatory responses, thereby impairing the tendon’s ability to maintain normal function [101,102]. Second, the mechanical loading experienced by the Achilles tendon differs across various types of physical activities, such as jumping and long-distance running. High-intensity loading, such as sprinting and jumping, can induce excessive intratendinous shear stress, leading to chronic inflammation and persistent pain [32]. In contrast, low-frequency repetitive loading, such as long-distance running, induces the expression of inflammatory genes including *IL-6*, *IL-1β*, and *Ptgs2* in the Achilles tendon, while prolonged running increases both the duration and extent of collagen degradation, resulting in the accumulation of degenerated collagen and the onset of tendinopathy [103]. Moreover, sustained excessive mechanical loading induces pathological alterations in the Achilles tendon. Although studies have shown that long-term mechanical loading increases Achilles tendon stiffness [41], downhill running for 4–8 weeks in mice resulted in reduced elastic modulus despite increased stiffness, rendering the tendon more susceptible to injury [104]. However, extrapolating these murine treadmill models to human chronic tendinopathy requires caution. Unlike the energy-storing capacity essential for human bipedal locomotion, the quadrupedal biomechanics of rodents impose fundamentally different strain magnitudes and regional stress distributions on the Achilles tendon. Clinical studies in patients with advanced-stage tendinopathy have confirmed the persistence of neovascularization within the Achilles tendon [105], suggesting that overuse induces abnormal angiogenesis that may be associated with pain. Although the presence of apoptosis in tendinopathy remains controversial, current evidence indicates that Achilles tendon overuse under high mechanical loading in rodent models induces apoptosis [106], thereby reducing the population of functional tenocytes required for ECM maintenance.

### 6.3. Insufficient Mechanical Loading and Achilles Tendon Degeneration

In contrast, insufficient mechanical loading induces Achilles tendon degeneration, involving alterations in biomechanical properties and structural characteristics of the tendon. Studies have demonstrated that inadequate mechanical loading of the Achilles tendon may lead to deterioration of its biomechanical properties and structure, thereby increasing the risk of tendon injury. First, insufficient mechanical loading results in reductions in the elastic modulus and stiffness of the Achilles tendon. Evidence indicates that Achilles tendon stiffness and elastic modulus are significantly lower in older adults than in younger individuals, which may be associated with insufficient mechanical loading due to age-related declines in physical activity and consequent reductions in tendon loading [107]. Second, insufficient mechanical loading may contribute to structural degeneration of the Achilles tendon. Research has shown that structural alterations of the Achilles tendon, including disorganized collagen fiber alignment and increased water content, are associated with insufficient mechanical loading. A study examining the effects of spaceflight on the mouse Achilles tendon demonstrated that microgravity markedly reduces mechanical loading, resulting in a significant decrease in collagen fiber diameter, potentially due to upregulation of collagen-degrading genes such as *MMP3* and *MMP13*, thereby impairing tendon function and increasing injury risk [108,109]. In addition, the inter-fascicular sliding capacity of the Achilles tendon decreases with aging, which may be attributed to load-induced increases in interfacial stiffness and a consequent elevation in injury risk [110]. Finally, a lack of mechanical loading may also affect the biological responses of the Achilles tendon. Studies have found that biological responses of the Achilles tendon, such as neovascularization, are influenced not only by load magnitude but also closely related to loading patterns, including high-frequency training [111]. Therefore, while avoiding insufficient loading is important, guiding favorable biological adaptations through scientifically designed training programs is essential for improving Achilles tendon health.

### 6.4. Metabolic Disorders and Secondary Tendinopathy

Systemic metabolic conditions, such as diabetes and hypercholesterolemia, critically precipitate “secondary tendinopathy” by altering the tendon’s biomechanical threshold. Clinical evidence shows that elevated HbA1c and cholesterol are associated with a twofold to threefold increased risk of tendinopathy [112]. These metabolically compromised tendons exhibit impaired ECM turnover and diminished physiological resilience, meaning routine mechanical loads can rapidly manifest as pathological overload. Therefore, mechanical loading strategies for patients with metabolic comorbidities must be meticulously adjusted, requiring much slower progression to prevent further tissue degeneration.

## 7. The Role of Mechanical Loading in Achilles Tendon Injury Repair

Similarly to other tendons, the Achilles tendon exhibits limited regenerative capacity after injury, and the resulting scar tissue fails to restore native biomechanical properties, with clinical data indicating that only 50% of patients regain pre-injury range of motion and load-bearing capacity [113]. Tendons originate from mesenchymal stem cells and are mechanosensitive, indicating that, like other musculoskeletal tissues, they are influenced by physical stimuli. Mechanical loading plays a critical role in Achilles tendon repair by modulating cellular activity, ECM remodeling, and biomechanical recovery. Appropriate mechanical stimulation promotes tendon regeneration, whereas excessive or improper loading inhibits healing and may induce fibrosis and abnormal fiber alignment.

First, appropriate mechanical loading facilitates tissue repair by stimulating collagen synthesis, enhancing cell proliferation, and improving tendon structural organization. Khayyeri et al. demonstrated that mechanical loading applied during the early healing phase (1–2 weeks postoperatively) most effectively improves collagen alignment and biomechanical properties, whereas exercise initiated at 4 weeks post-injury yields minimal benefit, highlighting the importance of timely rehabilitation [113]. Studies indicate that intermittent mechanical loading during the inflammatory phase significantly increases maximum force, stiffness, and peak stress during healing, and although early loading induces repetitive microdamage, this may improve healing by modulating inflammatory mediators such as IL-1β and TNF-α and promoting tissue remodeling and strengthening [114].

Second, mechanical loading regulates ECM homeostasis and influences gene expression related to collagen synthesis, inflammatory responses, and cellular differentiation. Interestingly, Hammerman et al. reported that mechanical loading during both the inflammatory phase (3 days) and early remodeling phase (14 days) rapidly alters gene expression, with loading during inflammation primarily upregulating inflammatory genes to enhance healing, whereas loading during early remodeling promotes healing mainly through ECM remodeling, fibroblast migration and proliferation, and collagen synthesis [115]. However, extrapolating these temporal findings from animal studies to human clinical practice requires critical caution. Rodent tenotomy models exhibit a highly robust intrinsic healing capacity, which does not fully replicate the protracted fibrotic scarring characteristic of human Achilles tendon injuries.

Although moderate mechanical loading is beneficial for healing, excessive or premature loading may lead to fibrosis, increased inflammation, and impaired mechanical properties of the Achilles tendon. Studies have demonstrated that premature mechanical loading after Achilles tendon injury induces macrophage activation and scar formation while simultaneously weakening tendon mechanical performance [116,117]. As highlighted by recent single-cell insights, this pathological shift is largely driven by intense fibroblast-immune cell crosstalk, where mechanically induced microdamage releases DAMPs that sustain inflammatory cascades, ultimately steering the tendon toward fibrosis rather than regeneration [118].

In summary, Achilles tendon healing is a complex process in which mechanical loading plays a crucial role in post-injury repair. Although current studies have elucidated some mechanisms by which mechanical loading influences the efficacy and rate of Achilles tendon healing, further research is needed to define the underlying mechanisms and identify the optimal timing and intensity of loading.

## 8. Clinical Applications and Practical Guidance

Scientific management of mechanical loading is crucial for preventing Achilles tendon injuries and promoting rehabilitation. Gradual load progression, avoidance of abrupt short-term load fluctuations, and individualized rehabilitation strategies can facilitate adaptive remodeling of the Achilles tendon, improve its mechanical properties, and reduce the risk of re-injury. Optimizing Achilles tendon health in both athletes and the general population requires the integration of advanced monitoring technologies and evidence-based training protocols.

### 8.1. Application of Load Management in the Prevention of Achilles Tendon Injury

The occurrence of Achilles tendon injury is closely associated with inappropriate distribution of mechanical loading, as both excessive loading and insufficient loading can impair tendon adaptability and structural integrity. Interestingly, recent studies have shown that Achilles tendon injuries are also common among intermittently active individuals who experience sudden load increases [119], often referred to as “weekend warriors.” Evidence indicates that moderate increases in loading enhance tenocyte activity and collagen synthesis, whereas excessive loading induces tenocyte apoptosis and collagen degradation, ultimately leading to Achilles tendon injury. Consequently, load management has gained increasing attention as an important preventive strategy in the field of sports-related injury prevention. Core strategies include progressive load increments, avoidance of abrupt load spikes, and provision of adequate recovery time [120,121], thereby reducing the risk of Achilles tendon injury. In addition, modern technologies such as wearable motion sensors can be used to accurately quantify the magnitude and frequency of loading experienced by the Achilles tendon during physical activity [122]. This approach likewise facilitates rational load management and reduces the risk of Achilles tendon injury. In fact, research on preventive strategies for Achilles tendinopathy remains limited, and future studies should continue to conduct basic research and clinical trials to identify effective approaches for preventing Achilles tendon injury [123].

### 8.2. Load Regulation Strategies in Rehabilitation Training

For acute Achilles tendon injuries, the optimal current treatment approach combines minimally invasive repair with accelerated functional rehabilitation [124]. However, a strict distinction must be drawn between post-rupture rehabilitation and the management of chronic tendinopathy. Post-rupture healing dictates that while early functional mobilization improves collagen alignment, premature high-strain loading can induce macrophage activation, excessive scar formation, and ultimately weaken the tendon. Therefore, post-rupture loading must prioritize low-strain motion to prevent adhesions. In contrast, for patients with Achilles tendinopathy, exercise represents the intervention with the highest level of evidence to date [98,125], aiming to apply progressive heavy resistance to reverse degenerative matrix changes and build load tolerance.

To achieve optimal functional recovery, current clinical consensus advocates for practical progression of loading, typically structured into four phases. Phase 1 focuses on symptom management and isometric loading to reduce excessive tendon strain, avoiding high-velocity and compressive loads [126]. Phase 2 transitions to progressive slow-velocity isotonic loading, such as heavy slow resistance (HSR) [127], continuing until a high load capacity is reached. In this context, studies have demonstrated that low-load blood flow restriction training (LL-BFR) and high-load resistance training can induce similar adaptations in tendon morphology and mechanical properties, which is particularly beneficial for patients who cannot tolerate high loads, as LL-BFR allows tendon adaptations to occur under relatively low mechanical loading [50,128]. Phase 3 introduces high-velocity energy storage and release exercises (e.g., plyometrics), mandating adequate inter-session recovery (e.g., 36 h) for ECM turnover [129]. Finally, Phase 4 relies on strict return-to-sport (RTS) criteria, integrating sport-specific movements like sprinting and rapid directional changes to mitigate re-injury risks [130].

During these progressive phases, load modulation involves not only the mechanical properties of the tendon but also adaptive changes in the neuromuscular system. A systematic review found that, in patients with chronic ankle instability, changes in brain structure and neural adaptations during rehabilitation are closely associated with ankle injury [131], suggesting that neuroplasticity should be considered in rehabilitation training. In addition, neuromuscular training has been shown to significantly improve biomechanical deficits of the knee joint in athletes after anterior cruciate ligament reconstruction, providing a reference for Achilles tendon rehabilitation by improving joint movement patterns and load distribution through progressive load levels and increasing training difficulty [132]. Furthermore, evidence indicates that Achilles tendon compliance has a greater influence on tendon loading than the degree of tendon structural twist, suggesting that rehabilitation should focus on changes in tendon compliance to more accurately predict muscle force and develop individualized rehabilitation strategies [133]. In summary, load modulation strategies must differentiate between injury types and comprehensively consider structured load progression, neuromuscular adaptations, and individual needs in order to achieve optimal functional recovery and safe return to sport.

## 9. Discussion

Through this review, we conducted a comprehensive analysis of the existing literature on the effects of mechanical loading on Achilles tendon function and structure, highlighting the major research trends and key findings in this field. Current evidence consistently indicates that the effects of mechanical loading on Achilles tendon structure and function depend on load magnitude and duration, with appropriate loading benefiting mechanical properties and structural characteristics, as well as promoting tendon healing [39,46,54,134]. But excessive or insufficient mechanical loading can result in structural damage to the Achilles tendon, reduced mechanical performance, and impaired post-injury healing [46,135,136]. Although previous studies have provided substantial insights into the effects of loading on Achilles tendon function and structure, and significant progress has been made in load management and tendon biomechanics, notable limitations and unresolved questions remain. Future research should elucidate the specific mechanisms of mechanical loading, investigate the application of load modulation in Achilles tendon repair, and optimize individualized load management strategies to improve prevention and rehabilitation outcomes for Achilles tendon injuries.

First, although existing studies suggest that mechanical loading may regulate tendon adaptation through cellular mechanotransduction, gene expression, and ECM metabolism, the underlying molecular mechanisms have not yet been fully elucidated. This knowledge gap may hinder our understanding of the diagnosis, treatment, and healing of Achilles tendon injuries, and future studies should focus on these issues to advance the field. Benefiting from emerging technologies such as proteomics, recent studies have shown that certain biomarkers can predict Achilles tendon healing outcomes. Current human Achilles healing data emphasize that while elevated levels of eukaryotic elongation factor-2 (eEF2) [137] and inter-alpha-trypsin inhibitor heavy chain 4 (ITIH4) [138] are associated with favorable healing, lower levels of the pro-inflammatory biomarker complement factor D (CFD) are predictive of more favorable healing outcomes and enhanced collagen type I expression [139]. These findings are valuable for predicting functional recovery in patients with Achilles tendon injuries; however, the specific molecular mechanisms and signaling pathways remain unclear, and addressing these issues may have important implications for treatment. In addition, in vitro cell culture models incorporating three-dimensional culture and bioreactor systems can better simulate the effects of mechanical loading on tenocytes, and the application of scRNA-seq and gene-editing techniques may further elucidate the role of mechanical signals in tendon adaptive remodeling. Furthermore, to bridge the gap between in vitro findings and clinical applications, preclinical in vivo models—particularly rat Achilles tendon injury models—are indispensable for evaluating translational therapies. Recent research has highlighted the therapeutic potential of combining biochemical modulation with physiological healing. For instance, studies utilizing rat Achilles tendon rupture models have demonstrated that the systemic administration of natural antioxidants, such as caffeic acid and quercetin, significantly improves both histopathological and biomechanical outcomes. Specifically, quercetin has been shown to enhance tendon healing by upregulating type I collagen expression, which directly translates to increased rupture strength, failure load, and stiffness [140]. Moreover, in the context of metabolic comorbidities, quercetin exerts profound protective effects against diabetic tendinopathy by downregulating NADPH oxidase (NOX) and interleukin-6 (IL-6) expression, thereby reducing reactive oxygen species (ROS) accumulation and apoptosis to preserve collagen fiber organization [141]. Similar biomechanical and structural benefits have also been observed with caffeic acid treatment [130]. Integrating such preclinical pharmacological interventions with precise mechanical loading protocols could represent a promising adjunctive strategy to optimize matrix remodeling and accelerate functional recovery.

Second, although load management is currently regarded as a core strategy in Achilles tendon rehabilitation, existing rehabilitation protocols still have notable limitations, as consensus has not been reached regarding the optimal type, intensity, and frequency of loading, and the potential benefits of emerging biomaterials for patients with Achilles tendon injuries remain to be further clarified. Therefore, future research should primarily focus on the following aspects. First, reconstruction of the tendon–bone interface is critical for functional recovery after Achilles tendon rupture, yet current studies lack direct comparisons of the effects of different exercise modalities during rehabilitation [142]. Accordingly, future investigations should examine how different loading patterns influence cellular behavior and matrix remodeling at the tendon–bone interface. Second, both premature and delayed initiation of rehabilitation training after Achilles tendon injury can affect functional recovery, making the optimal timing of load-based interventions an important topic for further study.

Third, advances in biomaterials and regenerative medicine suggest that tendon repair can be enhanced under controlled loading environments. Studies have shown that acellular polymer composite scaffolds can significantly improve Achilles tendon regeneration under mechanical stimulation [143], while biomimetic scaffolds and bioengineered materials can optimize tendon mechanical properties and cellular responses [144,145]. Recently, cutting-edge regenerative strategies have increasingly focused on actively modulating the local immune microenvironment to achieve scarless healing. For instance, novel melatonin-loaded Janus fibrous membranes designed to mimic the native paratenon have shown remarkable efficacy in scavenging reactive oxygen species (ROS) and preventing postoperative adhesions [146]. Furthermore, hybrid scaffolds utilizing a decellularized tendon matrix combined with the sustained release of stem cell-derived exosomes have demonstrated significant potential in driving macrophage polarization toward the anti-inflammatory M2 phenotype, thereby accelerating tendon-specific differentiation and structural alignment [147]. Future efforts should focus on developing mechanoresponsive biomaterials capable of dynamically adapting to different mechanical loading environments, which may substantially enhance repair outcomes. Because post-injury load management directly influences prognosis, strengthening research on the clinical application of load management strategies and adjunctive approaches may help accelerate functional recovery in patients with Achilles tendon injuries.

Finally, individualized management strategies for Achilles tendon loading have emerged as a major focus in recent years within the fields of sports medicine and rehabilitation medicine. By tailoring interventions to different populations and injury types, individualized management strategies can effectively optimize therapeutic outcomes and reduce the risk of re-injury. First, progressive load management is one of the core strategies in Achilles tendon rehabilitation, as current evidence indicates that load tolerance is low in the early rehabilitation phase, whereas the average loading magnitude increases markedly within the first two postoperative weeks [148]. Therefore, postoperative rehabilitation requires a gradual increase in loading to avoid secondary injury. However, how to more precisely quantify Achilles tendon load thresholds and how to prescribe progressive rehabilitation loading protocols remain issues requiring further investigation. Second, Achilles tendon rehabilitation is a long-term process that requires continuous monitoring and dynamic adjustment. Nevertheless, such studies remain relatively limited due to high costs and technological constraints. Existing studies have demonstrated that wearable sensors, three-dimensional motion capture, and ground reaction force measurement technologies can all enable real-time monitoring of Achilles tendon loading and support the development of personalized rehabilitation programs [149]. Future research should build on these advances by further developing relevant technologies and reducing costs to facilitate earlier clinical implementation. Third, external assistive technologies have also shown promise in Achilles tendon load management. Studies have shown that exoskeletons can significantly reduce Achilles tendon loading during loaded walking [150], while customized arch-support orthoses can effectively decrease additional Achilles tendon loads during running [151], thereby reducing the risk of Achilles tendinopathy. Further exploration of the feasibility of external assistive technologies and large-scale clinical trials are needed to validate their effectiveness and to provide effective preventive strategies for both athletes and the general population. In summary, individualized load management represents an important trend in the rehabilitation and prevention of Achilles tendon injuries, and future research should further explore precise load quantification and the development of intelligent management systems to advance this field.

## 10. Conclusions

By synthesizing the current literature, this review demonstrates that mechanical loading plays a crucial role in regulating Achilles tendon function and structure, influencing its biomechanical properties, tenocyte responses, and overall structural integrity. Existing studies indicate that appropriate mechanical loading maintains tendon health by increasing tendon stiffness and promoting collagen synthesis and ECM remodeling. In contrast, excessive or insufficient loading may induce maladaptive changes in tendon tissue, such as degenerative alterations and abnormal collagen organization. Although significant progress has been made, notable research gaps remain. At present, most studies focus primarily on the effects of short-term mechanical loading on the Achilles tendon, whereas long-term adaptive changes and their roles in injury prevention and rehabilitation require further investigation. Moreover, substantial inter-individual variability in responses to mechanical loading exists due to genetic and metabolic factors, the underlying mechanisms of which remain poorly understood. Future research should employ advanced imaging techniques and molecular biological approaches to elucidate the specific mechanisms underlying adaptive changes in the Achilles tendon. In parallel, integrating biomechanical modeling with experimental studies will facilitate the development of individualized strategies for Achilles tendon injury prevention and rehabilitation. By addressing these gaps, future studies will deepen our understanding of Achilles tendon adaptation mechanisms, thereby optimizing injury prevention, rehabilitation, and performance enhancement strategies, with the ultimate goal of improving the clinical management of Achilles tendon injuries.

## Figures and Tables

**Table 1:** The effects of various exercise modalities on stiffness, Young’s modulus, and CSA of the Achilles tendon.

Author, Year	Duration (Weeks)	Frequency (n/wk)	Intervention Characteristics (Intensity, Volume, Contraction/Exercise Type)	Stiffness	Modulus	CSA
Arampatzis et al., 2007 [48]	14	4	Low-strain/-intensity ISO vs. High-strain/-intensity ISO	↑	↑	↑
Bohm et al., 2014 [49]	14	4	High-strain/-intensity SSC vs. High strain/intensity ISO	↑	↑	↑
Centner et al., 2019 [50]	14	3	High-intensity, low-volume CON:ECC vs. low-load BFR	↑	↑	↑
Hirayama et al., 2017 [51]	12	3	High-intensity, high-volume SSC	↑	-	-
Fouré et al., 2009 [52]	8	2	Variable-intensity, variable-volume SSC	↑	-	-

Note: ISO—isometric. SSC—Stretch-Shortening Cycle. ECC. Eccentric. CON—Concentric. CON:ECC—concentric:eccentric. ↑—significant increase. -—no significant difference.

## Data Availability

No new data were created or analyzed in this study. Data sharing is not applicable to this article.
